# Early surgery with antibiotic medication was effective and efficient in treating pyogenic spondylodiscitis

**DOI:** 10.1186/s12891-021-04155-2

**Published:** 2021-03-18

**Authors:** Wei Guo, Min Wang, Guangfu Chen, Kuan-Hung Chen, Yong Wan, Bailing Chen, Xuenong Zou, Xinsheng Peng

**Affiliations:** 1 Department of Orthopedic Surgery, the First Affiliated Hospital, Sun Yat-sen University, Guangzhou, China; 2 Guangdong Provincial Key Laboratory of Orthopedics and Traumatology, Guangzhou, China; 3 Department of Spinal Surgery, Chancheng Central Hospital, Foshan, China

**Keywords:** Pyogenic spondylodiscitis, Laboratory test, Radiology, Surgery, Antibiotic

## Abstract

**Background:**

Pyogenic spondylodiscitis (PSD) is challenging to the orthopedist with regards to diagnosis and treatment. The present study was designed to assess and suggest the most indicative diagnostic method and evaluate the effect of surgery comprising of debridement, instrumentation and fusion in treating PSD.

**Methods:**

Seventy-six patients with PSD who underwent surgical intervention were retrospectively enrolled. Their medical documents, corrections of spinal alignment and improvements in neurological function were assessed. Surgical approaches were compared in lumbar surgeries regarding the improvements in lordotic angle and neurological function.

**Results:**

Elevated c-reactive protein (CRP) and erythrocyte sedimentation rate (ESR) were found in 77.6 and 71.1% patients respectively. Infectious lesions were found at lumbar (85.5%), cervical (10.5%) and thoracic (3.9%), ascertained with contrast-enhanced MRI. For lumbar patients, surgery was performed through the anterior (26.2%), posterior (49.2%) or combined approach (24.6%), and differences in improvement of lordosis and neurological function between each approach were insignificant. The pathogen was identified in 22.4% of the patients. Postoperative antibiotic therapy was managed against the result of susceptibility test, or empirically given to patients with negative cultures. All antibiotic therapy was initiated intravenously for 4–6 weeks and orally for 6 weeks.

**Conclusion:**

Elevated CRP and/or ESR, with focal hyper-intensity on contrast-enhanced MRI are suggestive of possible PSD. Surgical intervention comprising of debridement, short-segment instrumentation and fusion that early applied to the PSD patients followed by postoperative antibiotic therapy have demonstrated preferable outcomes, but require further study.

**The translational potential of this article:**

This article advocates early surgery to enable prompt diagnosis and treatment of PSD, and thus guarantee favorable outcomes for patients, as is shown in our study. In addition, different surgical approaches to the lesions were compared and discussed in this manuscript, but no differences in outcome between approaches were found. This suggests that thorough debridement should be prioritized over selection of surgical approach. In summary, this article has large translational potential to be applied clinically.

## Background

As one of the infectious spinal disorders, pyogenic spondylodiscitis (PSD) has increasing incidence globally [1, 2]. PSD could be caused by pathogens that hematogenously transferred from a distant infectious locale or directly spread from an adjacent site, involving the vertebrae and intervertebral discs [3]. The susceptible population are those with predisposing conditions, such as open wounds, operations, hemodialysis, oral infections, infective endocarditis, diabetes, HIV or other immunosuppressive diseases, so the presence of any of these factors increases the risks of developing PSD [4–6].

Since PSD lacks specific symptoms and signs, especially at the early stage, confirming the diagnosis is challenging to physicians. The infection-related symptoms and signs like fever and fatigue can be absent, and some spine-related symptoms and signs, such as local tenderness, restricted motion and even deformity or neurological impairments, may be confused with degenerated intervertebral disc or spondylolisthesis [7]. Therefore, the diagnosis of PSD largely relies on a combination of clinical investigations, laboratory tests, such as white blood cell (WBC), C-reactive protein (CRP) and erythrocyte sedimentation rate (ESR), and radiological evaluations like X-ray, computerized tomography (CT) and magnetic resonance imaging (MRI) [8]. However, personal judgement and experience of physicians to PSD plays a key role in timely diagnosis of the disease.

The conventional treatment strategy is conservative administration of sensitive antibiotics while surgical intervention is only initiated once the conservative treatment fails [9–11]. However, a recent study demonstrates that surgical treatment prior to conservative treatment resulted in fewer hospitalization days and more effective infection control [12]. Unfortunately, a consensus to guide the timing and approach of surgical intervention is still absent. To help physicians tackle this problem, we retrospectively investigated a case series of PSD. The diagnoses were achieved mainly depending on blood tests and radiological assessments. All patients directly underwent surgery and received post-operative antibiotic therapy. Both spinal alignment and neurological function significantly improved after the treatment. Moreover, in the lumbar cases, outcomes for different surgical approaches, i.e. anterior, posterior and combined, were similar regarding the improvements in lordotic angle and neurological function. This study suggests that this comprehensive strategy is effective and efficient in eradicating the infectious foci, improving symptoms, and restoring the alignment and stability of the spine.

## Methods

### Patients and clinical investigations

We retrospectively reviewed documents of the inpatients who were diagnosed with PSD and received surgeries in our department from Dec. 2006 to Apr. 2014. Seventy-six patients were enrolled, their corresponding medical documents including clinical presentation, blood test and radiological assessment were reviewed and analyzed. The study was approved by the Institutional Review Board (IRB) of the First Affiliated Hospital of Sun Yat-sen University.

The clinical presentation documented included general symptoms and signs such as pain, fever, fatigue and limited range of motion, as well as co-morbidities and predisposing conditions like diabetes, drug abuse, hepatitis and urological infection history. Neurological functions of each patient were evaluated according to the Kirkaldy-Willis Criteria (KWC) [13]. It describes the capability of patients returning to work or daily activity, and grades them by poor, fair, good and excellent. Body temperature, WBC, CRP and ESR were analyzed to assess extent and improvement of the infection. In radiological assessment, X-rays were used to measure Cobb angles to quantify the lordosis of cervical and lumbar or the kyphosis of thoracic. CT scans were used to clarifying the destruction of vertebral bodies and pedicles, and the contrast-enhanced MRIs for evaluating the range and degree of the infection. Measurements of Cobb angles were taken three times and the mean value recorded. All measurements were collected and compared pre- and post-operatively in order to determine the corresponding improvement.

### Surgeries

All the enrolled patients were treated with surgical intervention prior to antibiotic therapy, and the indications included intractable pain, spinal instability, neurological impairment, abscess formation and/or discitis found with MRI.

The surgical treatment comprised of a radical debridement of involved intervertebral disc and bone elements, an intervertebral fusion by autologous bone, an instrumentation with a titanium screw-and-rod system to stabilize the segment. The surgeries were performed through three approaches, i.e. the anterior approach, the posterior approach and the combination of both approaches, which were decided according to the location of infectious focus and the surgeon’s personal technique and judgment. For patients presented with psoas abscess, anterior approach is optimal to access the lesion; with epidural abscess, a posterior approach enables direct debridement; in patients with vertebral body erosion, anterior-posterior approach would be allow for excision of infected tissue anteriorly, while pedicle screws placed posteriorly offers rigid fixation. Anterior surgery in thoracic and lumbar regions was performed through retropleural or retroperitoneal approaches respectively, in which the infectious disc was debrided and a block of tricortical bone from the iliac crest was placed in the intervertebral space to strut and to fuse. The screw-and-rod system was fixed on the debrided vertebral bodies to restore alignment and stabilize the segment. The posterior surgery was performed through a midline incision followed by removal of the spinal process and laminae, so that the intervertebral disc could be debrided posteriorly and the bone graft could be placed into position. The segment was stabilized by pedicle screws inserted into the involved and the adjacent vertebrae. The combined approach was performed in a single stage, in which a Wiltse posterolateral spinal incision was employed to place the pedicle screws and an anterior debridement and tricortical bone graft placement was implemented.

The operating field was thoroughly rinsed with normal saline, H_2_O_2_ and betadine solution before closure of the wound. Drainage was utilized in all the patients, and continuous irrigation was employed to those who had a large amount of pus.

### Postoperative treatments and follow-ups

All the patients continued with postoperative antibiotic treatment for 10 to 12 weeks, with 4 to 6 weeks intravenously and 6 weeks orally. The antibiotics were prescribed against pathogen identified with surgical specimen culture and corresponding susceptibility test. Before the pathogen could be identified or when cultures were negative, broad-spectrum antibiotics such as cephalosporins were empirically administered. Symptoms like pain, and indicators of inflammation like CRP and ESR were regularly monitored to assess the effectiveness of antibiotics. External bracing was required for 3 months to assist ambulation. Outcomes were recorded at the 3-month, 6-month, and 1-year post-operation points and the final follow up, in which all the patient returned to the hospital and received physical and radiological examinations. All the outcomes from the follow ups were compared with preoperative findings.

### Statistics

Statistical analysis was performed with GraphPad Prism 8.0 software. The corrections of kyphosis or lordosis were analyzed using *one-way ANOVA.* The functional outcomes of KWC were analyzed using Kruskal-Wallis test. *P* < 0.05 was considered significant.

## Results

### Patients

There were 53 (69.7%) males and 23 (30.3%) females in the present study, and the mean age at the time of surgery was 55 years (19–85 years). The mean history of symptoms was 6.3 months (0.17–60 months), and intractable back pain and radiculopathy were presented as chief complaints in 75 (98.7%) and 38 (50%) patients respectively. Fever only occurred in 11 (14.5%) patients although it is a common sign for infection. Predisposal comorbidities were recorded in 21 (27.6%) patients, with diabetes being the most common (7 patients, %). There were 15 (19.7%) patients received physical therapy, but none reported acupuncture treatment history. Past surgical history was found in 3 (3.9%) patients: one had thyroidectomy and the other two had lumbar foraminotomy. Upon admission, the diagnosis of PSD was only confirmed in 6 (7.9%) patients, while 33 (43.4%) patients were considered having infection of unidentified causes while the rest were diagnosed with other diseases (Table 1).

**Table 1:** demographic data of the patients

Demographics	n. patients (%)
Gender	
male	53 (69.7%)
female	23 (30.3%)
Chief complains	
pain	75 (98.7%)
radiculopathy	38 (50%)
fever	11 (14.5%)
paraplegia	3 (3.9%)
Comorbidity and predisposing condition	
diabetes mellitus	7 (9.2%)
hypertension	4 (5.3%)
urinary tract infection	2 (2.6%)
gout	1 (1.3%)
rheumatoid arthritis	1 (1.3%)
Alzheimer’s	1 (1.3%)
chronic bronchitis	1 (1.3%)
Parkinson’s	1 (1.3%)
surgical history	3 (3.9%)
Initial diagnosis	
infection without specification	33 (43.4%)
tuberculosis	18 (23.7%)
pyogenic spondylodiscitis	6 (7.9%)
lower back pain	6 (7.9%)
lumbar disc herniation	5 (6.6%)
osseous erosion	4 (5.3%)
lumbar canal stenosis	1 (1.3%)
ankylosing spondylitis	1 (1.3%)
osteoporosis	1 (1.3%)
tumor	1 (1.3%)

### Laboratory parameters and culture yields

Values of WBC, CRP and ESR were elevated in 15 (19.7%), 59 (77.6%) and 54 (71.1%) preoperative patients respectively, and then gradually declined after surgical treatment. The elevations of CRP and ESR occurred together in most cases, however, 4 patients had elevated ESR with normal CRP, and 1 patient had elevated CRP with normal ESR. All the values returned to normal when the patient was discharged from hospital, and maintained at normal level until the final follow-up (Table 2).

**Table 2:** laboratory examinations for the patients

laboratory parameter	n. patients
Elevation in blood test	
WBC	15 (19.7%)
CRP	59 (77.6%)
ESR	54 (71.1%)
Microorganism	
no yields	59 (77.6%)
*Staphylococcus aureus*	7 (9.2%)
enterococcus faecalis	2 (2.6%)
ochrobactrum anthropi	1 (1.3%)
*Staphylococcus epidermidis*	1 (1.3%)
citrobacter braakii	1 (1.3%)
staphylococcus hominis	1 (1.3%)
acinetobacter baumannii	1 (1.3%)
staphylococcus xylosus	1 (1.3%)
micrococcus varians	1 (1.3%)
MRSCoN	1 (1.3%)

*MRSCoN* methicillin resistant coagulase negative staphylococci

*WBC* white blood cell

*CRP* c-reactive protein

*ESR* erythrocyte sedimentation rate

Pathogens were only found in 17 (22.4%) patients, among which *Staphylococcus aureus* was the most common type (7 / 17, 41.2%). In contrast, in majority of the patients (59, 77.6%) the microbial culture failed to yield detectable microorganisms (Table 2).

### Surgeries and spinal alignments

The infections occurred mainly at the lumbar region which were found in 65 (85.5%) patients, and then at cervical and at thoracic regions in 8 (10.5%) and 3 (3.9%) patients respectively (Table 3). Moreover, the most affected segment was L4/5 (26, 34.2%).

**Table 3:** the distribution of cases of each spinal region and the corresponding average improvement in alignment and functional outcome

Segment	n. of cases^a^	The Cobb angle (°, mean ± STD)			High KWC (n. of cases^b^)		
		pre-operative	post-operative	correction	pre-operative	post-operative	improvement
Cervical	8 (10.5%)	5.8 ± 3.6	14.4 ± 13.8	8.5 ± 15.2	1 (12.5%)	7 (87.5%)	6
C3/4	3 (3.9%)						
C4/5	3 (3.9%)						
C6/7	2 (2.6%)						
Thoracic	3 (3.9%)	31.6 ± 7.7	32.3 ± 9.1	0.7 ± 1.4	0	2 (66.7%)	2
T10/11	1 (1.3%)						
T11/12	2 (2.6%)						
Lumbar	65 (85.5%)	33.1 ± 11.6	37.6 ± 10.2	4.5 ± 9.6	12 (18.5%)	56 (86.2%)	44
T12/L1	1 (1.3%)						
L1/2	6 (7.9%)						
L2/3	5 (6.6%)						
L3/4	14 (18.4%)						
L3–5	3 (3.9%)						
L3-S1	1 (1.3%)						
L4/5	26 (34.2%)						
L4-S1	1 (1.3%)						
L5/S1	8 (10.5%)						

^a^, the percentage in brackets is for all the 76 patients

^b^, the percentage in brackets is for patients with the corresponding surgical region

High KWC means *good* or *excellent*

At final follow-up, the mean lateral Cobb angle improved from 5.8° ± 3.6° to 14.4° ± 13.8° in cervical patients, with a correction of 8.5° ± 15.2°. Thoracic patients improved from 31.6° ± 7.7° to 32.3° ± 9.1°, with a correction of 0.7° ± 1.4°. Lumbar patients improved from 33.1° ± 11.6° to 37.6° ± 10.2°, with a correction of 4.5° ± 9.6° (Table 3).

**Fig. 1:**
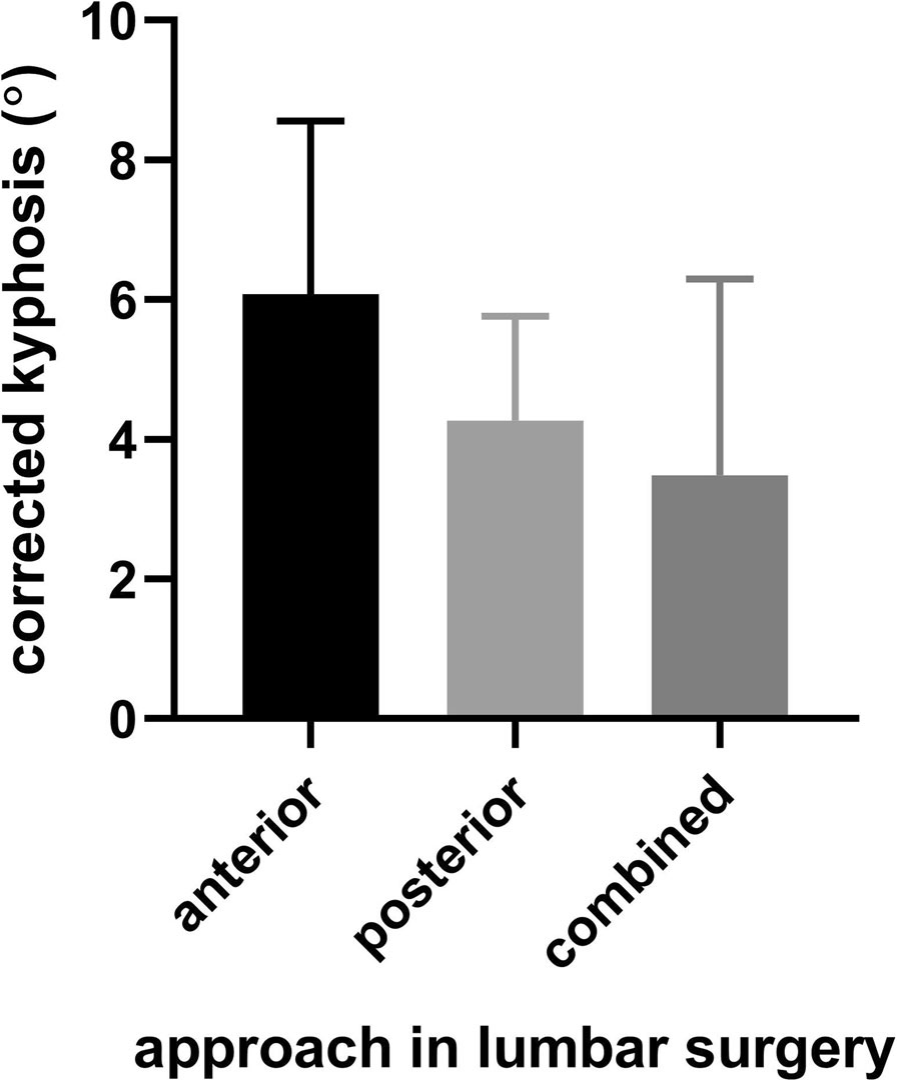
The mean corrected kyphotic angles of different approaches in lumbar surgery

Data from the lumbar patients were further analyzed to compare the improvements resulting from different surgical approaches. As shown in Table 4, the corrected lordotic angle was 6.1° ± 10.3°, 4.3° ± 8.4° and 3.5° ± 11.2° in patients treated via the anterior, posterior and combined approach respectively. However, the corrections were insignificant between the three approaches (*p* > 0.05) (Fig. 1).

**Table 4:** improvements of lordosis and functional outcomes in lumbar patients

Approach	n. of cases^a^	The Cobb angle (°, mean ± STD)			High KWC (n. of cases^b^)		
		pre-operative	post-operative	correction	pre-operative	post-operative	improved
anterior	17 (26.2%)	33.0 ± 12.3	39.1 ± 15.5	6.1 ± 10.3	5 (29.4%)	16 (94.1%)	11 (64.7%)
posterior	32 (49.2%)	31.3 ± 10.3	35.5 ± 7.1	4.3 ± 8.4	4 (12.5%)	28(87.5%)	24 (75%)
combined	16 (24.6%)	36.8 ± 13.2	40.2 ± 8.2	3.5 ± 11.2	3 (18.6%)	12 (75%)	9 (56.3%)

^a^, the percentage in brackets is only for the 65 lumbar patients

^b^, the percentage in brackets is only for patients with the corresponding approach, i.e. 17, 32, 16 respectively

High KWC means *good* or *excellent*

### Outcome evaluations

**Fig. 2:**
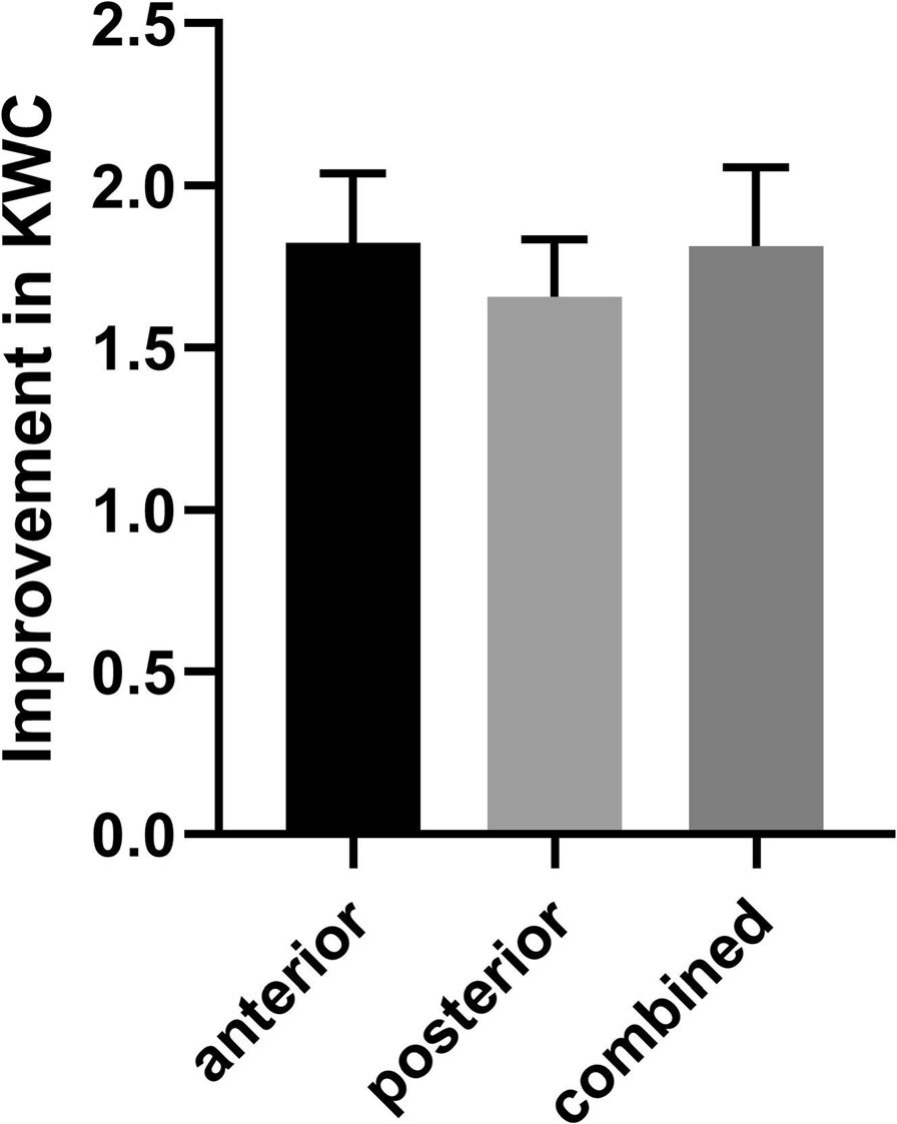
The improvement of KWC in patients with different surgical approaches in lumbar surgery

Neurological function was evaluated by KWC grading, and the Improvement was determined by achieving a grade of “good” and “excellent” at pre-treatment and final follow-up. In cervical cases, the rate increased from 1 (12.5%) to 7 (87.5%), while in thoracic from 0 to 2 (66.7%) and in lumbar from 12 (18.5%) to 56 (86.2%) (Table 3). Moreover, in lumbar surgeries the improvement resulted from anterior, posterior and combined approach was 5 (7.7%) to 16 (24.6%), 4 (6.2%) to 28 (43.1%) and 3 (4.6%) to 12 (18.5%) respectively (Table 4). The differences between the lumbar approaches were insignificant (Fig. 2).

### Complications

Delayed wound healing occurred in 3 patients, but with daily dressing change, all the wounds healed within 1 month after surgery. Graft donor site pain was reported in two patients during follow-up, with a visual analogue scale (VAS) score of 1 and 2 respectively. Revision surgery was conducted in 3 patients because of recurrent infection. The recurring infections were found at adjacent segments in 2 of the patients 8 months after the first treatment, and then they received a revision surgery adopting the same strategy. In the other patient the recurrence was found at the original site 2 years after the initial surgery, so all internal fixatives were extracted with the infected tissue removed in reoperation, and an external bracing was used to stabilize the spine until intervertebral fusion was confirmed with radiography. All 3 patients returned to work and participated in regular social activities by the 6–8th post-operative month. Until final follow-up, neither instrumental failure nor non-fusion were reported in all patients.

None of the patients complained of recurrent PSD symptoms following post-surgical antibiotic treatment at the final follow up, and no signs of recurrence were found in the accompanied blood tests and radiological examinations.

## Discussion

Although PSD is an infection, it does not always demonstrate infectious symptoms like high body temperature or elevated blood WBC during the early stage. In our study, only 13.2% of the patients presented with fever preoperatively, with a mean WBC of 8.27 * 10^9^/L. In contrast, elevated CRP and ESR were found in 77.6 and 71.1% of pre-operative patients respectively. These results are similar to those in other researches where the percentage of patients presenting with fever ranged from 25 to 62.5% [14–16] and the mean WBC was around 9.28 to 10.15 * 10^9^/L [17, 18], while CRP and ESR were elevated in more than 60% of the PSD patients [19, 20]. It is notable that elevation of just CRP or ESR may also imply the presence of infection. Our findings suggest that physicians should be aware of possible PSD in patients who complain about significant back pain and limited range of motion while CRP and/or ESR are elevated, even if leukocytosis or high body temperature is absent [21]. Moreover, the continuous monitor of the CRP and ESR postoperatively until the end of the antibiotic regime could help the physicians determine how the patients respond to the treatment [22, 23].

MRI has been widely recognized as the golden standard for imaging of spinal infections [24]. With contrast-enhancement, MRI is able to display trivial changes in vertebral body and in intervertebral disc even at the early stage of PSD, which helps to clarify the location and extent of the inflammatory focus and differentiate the disease from non-infectious changes [25–28]. However, if severe bone destruction was observed on the MRI, we propose the use of CT scans to clarify the extent of the destruction and evaluation for screw insertion.

Due to the deep location of the infectious loci, fluoroscopic or CT-guided percutaneous biopsy is often used in patients suspected of PSD. However, the harvested tissue are often too small to guarantee diagnostic accuracy, resulting in negative cultures, especially from patients with antecedent antibiotic treatment [29, 30]. Percutaneous biopsy also yields unsatisfactory results for histological identification of whether the lesion was a specific infection, such as tuberculosis, or a non-specific infection such as PSD. For example, one patient in our study underwent a preoperative CT-guided biopsy from which the aspirated sample suggested only PSD histologically, but the final surgical specimen was histologically identified as a tubercular with pyogenic co-infection. Moreover, performing percutaneous biopsy causes extra invasive procedures, which would potentiate puncture-related complications and discomforts of the patient [31, 32]. Based on these facts, we recommend a surgical approach upon diagnosis through clinical investigation, laboratory tests and radiological assessment. Rapid identification of the pathogen via surgical sample should be prioritized over biopsy by which the sufficient volume of tissue allows for reliable histological findings and greater chances of identifying the pathogen.

**Fig. 3:**
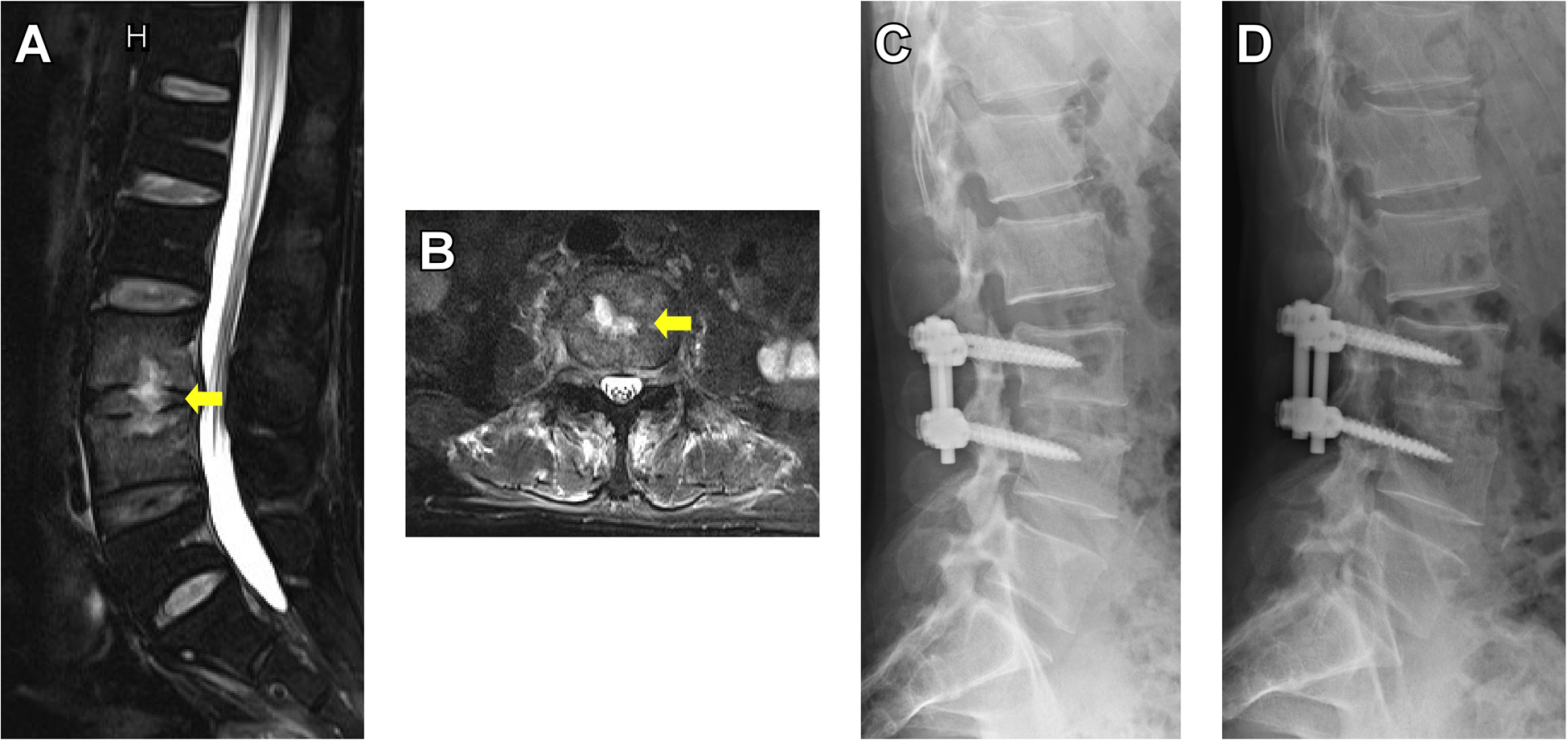
Pre- and post-operative radiological images of a 54-year-old male with intractable low back pain and limited range of motion of waist. **a**, at admission, endplate erosion and abscess (yellow arrow) at L3/4 intervertebral were disc seen on T2-weighted MRI. **b**, the abscess (yellow arrow) localized at the center of the disc. **c**, postoperative X-ray filming at the 1-month follow up. **d**, X-ray filming in 2 years after treatment

Once the diagnosis of PSD is confirmed, treatment should be initiated immediately. Conservative treatment is commonly the primary management of PSD and has been widely proven to be effective in controlling early infection. However, when conservative treatments fail or significant symptoms develop, such as significant bone destruction, neurological impairment and spinal instability, surgical intervention should be implemented as soon as possible to eliminate the infection and restore the alignment of the spine (Fig. 3) [23, 24, 33–35]. For patients who are elderly and predisposed with risk factors like hemodialysis, diabetes, infective endocarditis, etc., surgical intervention should also be initiated early to avoid prolonged hospitalization and consequent in-hospital mortality [2, 36]. However, it remained vague that when the early surgery should be conducted due to the lack of associated researches, therefore further studies are still needed.

**Fig. 4:**
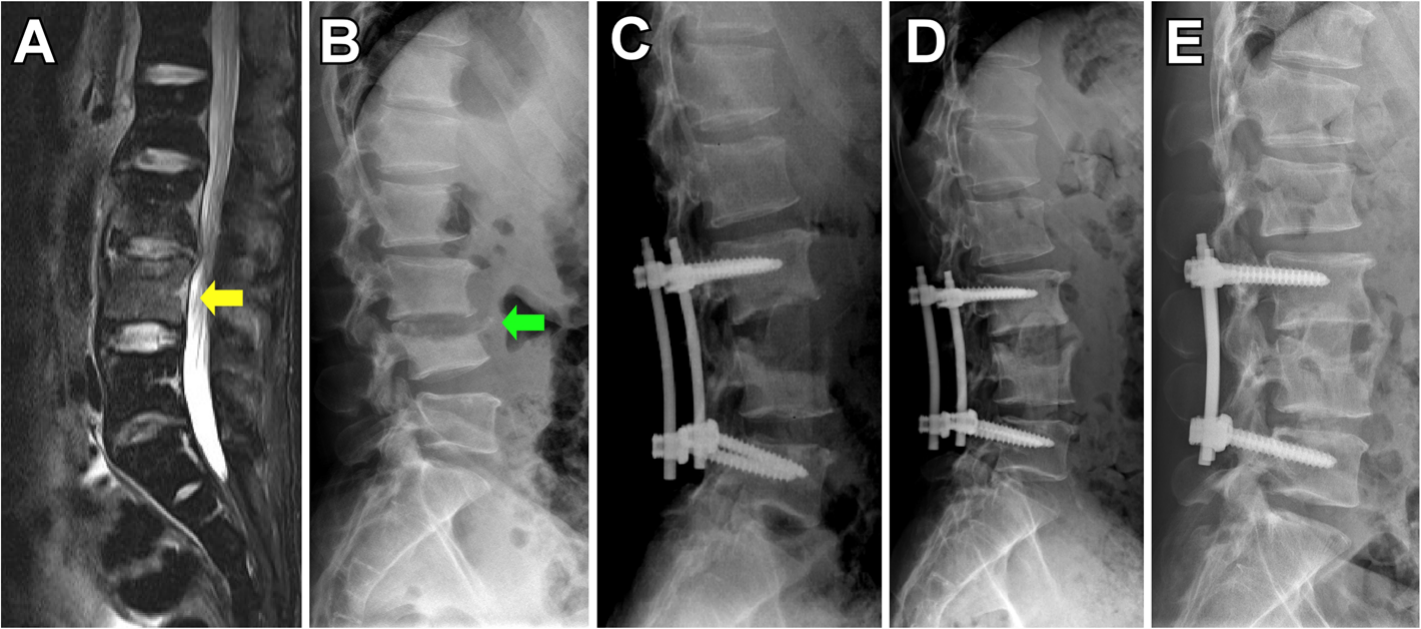
Pre- and post-treatment radiological images of a 45-year-old male with intractable low back pain and limited range of motion of waist. **a**, at first consultation, intensified signal at L3, L4 vertebral bodies were seen on T2-weighted MRI (yellow arrow). **b**, treated for 6 weeks with intravenous antibiotic therapy alone, the symptoms deteriorated and L4 body destructed (green arrow). **c**, instant X-ray filming after a combined surgical approach of posterior instrumentation and anterior debridement and fusion. **d**, postoperative X-ray filming at the 6-month follow up. E, postoperative X-ray filming at the 12-month follow up

The aims of early surgical treatment are to provide 1) early debridement and elimination of bacterial load, 2) rigid internal fixation that guarantees early ambulation and intervertebral fusion to restores spinal stability, 3) accurate acquisition of specimen of the infection for bacterial culture and histological examination. Therefore, we applied a surgical procedure in the present study where debridement, autograft and internal fixation were implemented thoroughly in the early stage. There were three surgical approaches involved in lumbar operations, and the outcomes were almost equal in terms of improvement in KWC score and kyphotic angle. This result was similar to previous studies where either approach was comparable to one of the others regarding the elimination of infection, improvement of symptoms, intervertebral fusion rates and restoration of spinal alignment [37, 38]. Nonetheless, each of the three approaches has its own advantages. For example, the posterior approach enabled profound drainage of the epidural abscess and meanwhile enables rigid fixation via pedicle screws, while the anterior approach minimizes damage to paraspinal muscles and bone structures [39–41]. The combined approach have the benefits of both approaches and therefore allows: 1) direct debridement of the infectious focus, 2) better sagittal deformity correction, and 3) short-segment fixation which was least interfering to spinal motion (Fig. 4) [38, 42, 43]. It is notable that the short-segment fixation along with intervertebral fusion could offer as good outcome in the correction of lordosis and improvement of neurological function as long-segment fixation would, even if the vertebral bodies were eroded or destroyed [44–46].

Post-operative antibiotics play an important role in treating PSD. In the present study, we select antibiotic agents according to bacterial cultures and susceptibility tests, if available. For example, vancomycin was used in the patients whose culture yield *methicillin-resistant Staphylococcus aureus* (MRSA). However, as aforementioned the rate of positive culture is low, so in most cases the antibiotic therapy was selected depending on history of the primary infection, macro view of the lesion and microscopic findings of the specimen. Routinely we start with empirical use of broad-spectrum antibiotics such as cephalosporin. If it failed to control the elevated CRP and ESR, carbapenem such as imipenem or meropenem was prescribed. The antibiotics were utilized intra-venously for no less than 4–6 weeks*,* followed orally for 6 weeks, until the values of CRP and ESR returned to normal [3, 47].

### Strengths and limitations

This study summarized our experience in diagnosing and treating PSD, all of which were concluded from and verified in our clinical practice. In the long-term follow ups, low recurrence rate of the PSD reaffirmed the effectiveness of this strategy. The advance that our study represents is the urgency of surgery in the treatment of PSD over conventional treatment strategy as it eliminates the infection and identifies the pathogen.

This study has some limitations that should be addressed. Firstly, as a retrospective study, the level of evidence is relatively low. In future studies a control group should be set up so that a thorough comparison and a stronger conclusion could be made. Secondly, despite a larger sample size compared to existing studies, this analysis is unable to justify a specific criterion for early surgical intervention. In the future, more comprehensive studies are still needed to define a treatment protocol for PSD.

## Conclusion

Surgery in the early stage of PSD, as shown in the present study, facilitates faster recovery and better outcomes due to timely use of selective antibiotics, as surgery specimens yields higher rates of positive culture and removes bacterial load. Furthermore, early surgical intervention restores spinal stability and prevents deformity due to erosion of the vertebrae and discs.

## Data Availability

The datasets generated and analysed during the current study are not publicly available due to hospital regulations, but can be made available from the corresponding author upon reasonable request.

## Abbreviations

PSD: Pyogenic spondylodiscitis
WBC: White blood cell counting
CRP: C-reactive protein
ESR: Erythrocyte sedimentation rate
CT: Computed tomography
MRI: Magnetic resonance imaging

## References

[1] Kehrer M, Pedersen C, Jensen TG, Lassen AT (2014). Increasing incidence of pyogenic spondylodiscitis: a 14-year population-based study. J Inf Secur.

[2] Akiyama T, Chikuda H, Yasunaga H, Horiguchi H, Fushimi K, Saita K. Incidence and risk factors for mortality of vertebral osteomyelitis: a retrospective analysis using the Japanese diagnosis procedure combination database. BMJ Open. 2013;3(3):e002412. PMID: 23533214. 10.1136/bmjopen-2012-002412. 10.1136/bmjopen-2012-002412 PMC3612742 23533214

[3] Rutges JP, Kempen DH, van Dijk M, Oner FC (2016). Outcome of conservative and surgical treatment of pyogenic spondylodiscitis: a systematic literature review. Eur Spine J.

[4] Kim CJ, Kim UJ, Kim HB, Park SW, Oh MD, Park KH, Kim NJ (2016). Vertebral osteomyelitis caused by non-tuberculous mycobacteria: predisposing conditions and clinical characteristics of six cases and a review of 63 cases in the literature. Infect Dis (Lond).

[5] Muzii VF, Mariottini A, Zalaffi A, Carangelo BR, Palma L (2006). Cervical spine epidural abscess: experience with microsurgical treatment in eight cases. J Neurosurg Spine.

[6] Courjon J, Lemaignen A, Ghout I, Therby A, Belmatoug N, Dinh A, Gras G, Bernard L (2017). Pyogenic vertebral osteomyelitis of the elderly: characteristics and outcomes. PLoS One.

[7] Skaf GS, Domloj NT, Fehlings MG, Bouclaous CH, Sabbagh AS, Kanafani ZA, Kanj SS (2010). Pyogenic spondylodiscitis: an overview. J Infect Public Health.

[8] Lener S, Hartmann S, Barbagallo GMV, Certo F, Thome C, Tschugg A (2018). Management of spinal infection: a review of the literature. Acta Neurochir.

[9] Babouee Flury B, Elzi L, Kolbe M, Frei R, Weisser M, Scharen S, Widmer AF, Battegay M (2014). Is switching to an oral antibiotic regimen safe after 2 weeks of intravenous treatment for primary bacterial vertebral osteomyelitis?. BMC Infect Dis.

[10] Pola E, Logroscino CA, Gentiempo M, Colangelo D, Mazzotta V, Di Meco E, Fantoni M (2012). Medical and surgical treatment of pyogenic spondylodiscitis. Eur Rev Med Pharmacol Sci.

[11] Butler JS, Shelly MJ, Timlin M, Powderly WG, O'Byrne JM (2006). Nontuberculous pyogenic spinal infection in adults: a 12-year experience from a tertiary referral center. Spine (Phila Pa 1976).

[12] Tsai TT, Yang SC, Niu CC, Lai PL, Lee MH, Chen LH, Chen WJ (2017). Early surgery with antibiotics treatment had better clinical outcomes than antibiotics treatment alone in patients with pyogenic spondylodiscitis: a retrospective cohort study. BMC Musculoskelet Disord.

[13] Bertilson BC, Bring J, Sjoblom A, Sundell K, Strender LE (2006). Inter-examiner reliability in the assessment of low back pain (LBP) using the Kirkaldy-Willis classification (KWC). Eur Spine J.

[14] Kim CJ, Song KH, Jeon JH, Park WB, Park SW, Kim HB, Oh MD, Choe KW, Kim NJ (2010). A comparative study of pyogenic and tuberculous spondylodiscitis. Spine (Phila Pa 1976).

[15] Kapsalaki E, Gatselis N, Stefos A, Makaritsis K, Vassiou A, Fezoulidis I, Dalekos GN (2009). Spontaneous spondylodiscitis: presentation, risk factors, diagnosis, management, and outcome. Int J Infect Dis.

[16] Bettini N, Girardo M, Dema E, Cervellati S (2009). Evaluation of conservative treatment of non specific spondylodiscitis. Eur Spine J.

[17] Griffith-Jones W, Nasto LA, Pola E, Stokes OM, Mehdian H (2018). Percutaneous suction and irrigation for the treatment of recalcitrant pyogenic spondylodiscitis. J Orthop Traumatol.

[18] Heyer CM, Brus LJ, Peters SA, Lemburg SP (2012). Efficacy of CT-guided biopsies of the spine in patients with spondylitis--an analysis of 164 procedures. Eur J Radiol.

[19] Kaya S, Ercan S, Kaya S, Aktas U, Kamasak K, Ozalp H, Cinar K, Duymus R, Boyaci MG, Akkoyun N, Eskazan AE, Temiz H (2014). Spondylodiscitis: evaluation of patients in a tertiary hospital. J Infect Develop Countries.

[20] Shiban E, Janssen I, Wostrack M, Krieg SM, Horanin M, Stoffel M, Meyer B, Ringel F (2014). Spondylodiscitis by drug-multiresistant bacteria: a single-center experience of 25 cases. Spine J.

[21] Seigel TA, Cocchi MN, Salciccioli J, Shapiro NI, Howell M, Tang A, Donnino MW (2012). Inadequacy of temperature and white blood cell count in predicting bacteremia in patients with suspected infection. J Emerg Med.

[22] Sheikh AF, Khosravi AD, Goodarzi H, Nashibi R, Teimouri A, Motamedfar A, Ranjbar R, Afzalzadeh S, Cyrus M, Hashemzadeh M (2017). Pathogen identification in suspected cases of pyogenic spondylodiscitis. Front Cell Infect Microbiol.

[23] Noh SH, Zhang HY, Lim HS, Song HJ, Yang KH (2017). Decompression alone versus fusion for pyogenic spondylodiscitis. Spine J.

[24] Cheung WY, Luk KD (2012). Pyogenic spondylitis. Int Orthop.

[25] Li T, Li W, Du Y, Gao M, Liu X, Wang G, Cui H, Jiang Z, Cui X, Sun J (2018). Discrimination of pyogenic spondylitis from brucellar spondylitis on MRI. Medicine.

[26] Moritani T, Kim J, Capizzano AA, Kirby P, Kademian J, Sato Y (2014). Pyogenic and non-pyogenic spinal infections: emphasis on diffusion-weighted imaging for the detection of abscesses and pus collections. Br J Radiol.

[27] Shapiro R (1986). In vitro fertilisation. Whatever happened to Warnock?. Nurs Times.

[28] Diehn FE (2012). Imaging of spine infection. Radiol Clin N Am.

[29] Rimondi E, Rossi G, Bartalena T, Ciminari R, Alberghini M, Ruggieri P, Errani C, Angelini A, Calabro T, Abati CN (2011). Percutaneous CT-guided biopsy of the musculoskeletal system: results of 2027 cases. Eur J Radiol.

[30] Lopes Floro K, Munckhof W, Coucher J (2018). Retrospective review of CT-guided intervertebral disc biopsies performed at a tertiary referral Centre for suspected osteodiscitis. J Med Imaging Radiat Oncol.

[31] Lis E, Bilsky MH, Pisinski L, Boland P, Healey JH, O'Malley B, Krol G (2004). Percutaneous CT-guided biopsy of osseous lesion of the spine in patients with known or suspected malignancy. AJNR Am J Neuroradiol.

[32] Arsov C, Rabenalt R, Quentin M, Hiester A, Blondin D, Albers P, Antoch G, Schimmoller L (2016). Comparison of patient comfort between MR-guided in-bore and MRI/ultrasound fusion-guided prostate biopsies within a prospective randomized trial. World J Urol.

[33] Schomacher M, Finger T, Koeppen D, Suss O, Vajkoczy P, Kroppenstedt S, Cabraja M (2014). Application of titanium and polyetheretherketone cages in the treatment of pyogenic spondylodiscitis. Clin Neurol Neurosurg.

[34] Pola E, Autore G, Formica VM, Pambianco V, Colangelo D, Cauda R, Fantoni M (2017). New classification for the treatment of pyogenic spondylodiscitis: validation study on a population of 250 patients with a follow-up of 2 years. Eur Spine J.

[35] Nickerson EK, Sinha R (2016). Vertebral osteomyelitis in adults: an update. Br Med Bull.

[36] Valancius K, Hansen ES, Hoy K, Helmig P, Niedermann B, Bunger C (2013). Failure modes in conservative and surgical management of infectious spondylodiscitis. Eur Spine J.

[37] Vcelak J, Chomiak J, Toth L (2014). Surgical treatment of lumbar spondylodiscitis: a comparison of two methods. Int Orthop.

[38] Linhardt O, Matussek J, Refior HJ, Krodel A (2007). Long-term results of ventro-dorsal versus ventral instrumentation fusion in the treatment of spondylitis. Int Orthop.

[39] Than KD, Wang AC, Rahman SU, Wilson TJ, Valdivia JM, Park P, La Marca F (2011). Complication avoidance and management in anterior lumbar interbody fusion. Neurosurg Focus.

[40] An KC, Kim JY, Kim TH, Kim JS, Park DH, Kim JG, Sung TW (2012). Posterior lumbar interbody fusion using compressive bone graft with allograft and autograft in the pyogenic discitis. Asian spine journal.

[41] Zhou B, Kang YJ, Chen WH. Continuous Epidural Irrigation and Drainage Combined with Posterior Debridement and Posterior Lumbar Inter-Body Fusion for the Management of Single-Segment Lumbar Pyogenic Spondylodiscitis. Surg Infect (Larchmt). 2020;21(3):262-7. PMID: 31647391. 10.1089/sur.2019.038. 10.1089/sur.2019.038 31647391

[42] Si M, Yang ZP, Li ZF, Yang Q, Li JM (2013). Anterior versus posterior fixation for the treatment of lumbar pyogenic vertebral osteomyelitis. Orthopedics.

[43] Tan T, Donohoe TJ, Huang MS, Rutges J, Marion T, Mathew J, Fitzgerald M, Tee J (2020). Does combined anterior-posterior approach improve outcomes compared with posterior-only approach in traumatic thoracolumbar burst fractures?: a systematic review. Asian Spine J.

[44] Liu Z, Zhang P, Zeng H, Xu Z, Wang X (2018). A comparative study of single-stage transpedicular debridement, fusion, and posterior long-segment versus short-segment fixation for the treatment of thoracolumbar spinal tuberculosis in adults: minimum five year follow-up outcomes. Int Orthop.

[45] Liang Q, Wang Q, Sun G, Ma W, Shi J, Jin W, Shi S, Wang Z (2018). Five-year outcomes of posterior affected-vertebrae fixation in lumbar tuberculosis patients. J Orthop Surg Res.

[46] Kim YM, Choi SM (2016). Posterior only approach for lumbar pyogenic spondylitis with short instrumentation and prolonged suction drainage. Spine (Phila Pa 1976).

[47] McHenry MC, Easley KA, Locker GA (2002). Vertebral osteomyelitis: long-term outcome for 253 patients from 7 Cleveland-area hospitals. Clin Infect Dis.

